# Development and Validation of a Differentiating Infected from Vaccinated Animals (DIVA) Enzyme-Linked Immunosorbent Assay (ELISA) Strategy for Distinguishing Between Hendra-Infected and Vaccinated Horses

**DOI:** 10.3390/v17030354

**Published:** 2025-02-28

**Authors:** Leanne McNabb, Amy McMahon, Ezana Getachew Woube, Kalpana Agnihotri, Axel Colling, Christopher C. Broder, Indre Kucinskaite-Kodze, Rasa Petraityte-Burneikiene, Timothy R. Bowden, Kim Halpin

**Affiliations:** 1 CSIRO Australian Centre for Disease Preparedness (ACDP), 5 Portarlington Road, Geelong, VIC 3220, Australia; amy.mcmahon@csiro.au (A.M.); kalpana.agnihotri@csiro.au (K.A.); axel.colling@csiro.au (A.C.); timothy.bowden@csiro.au (T.R.B.); kim.halpin@csiro.au (K.H.); 2 Private Consultant, Melbourne, VIC 3000, Australia; 3 Department of Microbiology and Immunology, Uniformed Services University, Bethesda, MD 20814, USA; christopher.broder@usuhs.edu; 4 Institute of Biotechnology, Life Sciences Centre, Vilnius University, LT-01513 Vilnius, Lithuania; indre.kodze@bti.vu.lt (I.K.-K.); rasa.burneikiene@bti.vu.lt (R.P.-B.)

**Keywords:** Hendra virus, Henipavirus, serology, DIVA, ELISA

## Abstract

Hendra virus (HeV) is a bat-borne zoonotic agent which can cause a severe and highly fatal disease and can be transferred from animals to humans. It has caused over 100 deaths in horses since it was discovered in 1994. Four out of seven infected humans have died. Since the release of the HeV vaccine (Equivac^®^ HeV Hendra Virus Vaccine for Horses, Zoetis Australia Pty Ltd., Rhodes, NSW 2138) in Australia, there has been an urgent requirement for a serological test for differentiating infected from vaccinated animals (DIVA). All first-line diagnostic serological assays at the Australian Centre for Disease Preparedness (ACDP) incorporate recombinant HeV soluble G glycoprotein (sG) as the antigen, which is also the only immunogen present in the Equivac^®^ HeV vaccine. Problems therefore arose in that antibody testing results were unable to distinguish between prior vaccination or infection with HeV. This study describes the development of a HeV DIVA ELISA strategy using recombinant sG and HeV nucleoprotein (N), paired with specific monoclonal antibodies in a competition ELISA format. The validation of this assay strategy was performed using a positive cohort of 19 serum samples representing post-infection sera, a negative cohort of 1138 serum samples representing horse sera collected pre-vaccine release and a vaccination cohort of 502 serum samples from horses previously vaccinated with Equivac^®^ HeV vaccine. For the sG glycoprotein, the diagnostic sensitivity (DSe) was 100.0% (95% CI: 99.3–100.0%) and diagnostic specificity (DSp) 99.91% (95% CI: 99.5–100.0%), using a percentage inhibition cut-off value of >36, whereas for the N protein, DSe was 100.0% (95% CI: 82.4–100.0%) and DSp 100.0% (95% CI: 99.7–100.0%), using a percentage inhibition cut-off value of >49. Taken together, these results demonstrate that the HeV DIVA ELISA strategy developed here is now an essential and critical component of the testing algorithm for HeV serology testing in Australia.

## 1. Introduction

Hendra virus (HeV) is a highly pathogenic zoonotic paramyxovirus, within the genus *Henipavirus*, capable of infecting a diverse array of mammalian species including humans. HeV was discovered as the causative agent of an outbreak of severe disease in horses and humans in 1994, in the Brisbane suburb of Hendra, Queensland, Australia [1]. The natural hosts of HeV are flying foxes (fruit bats) from the genus *Pteropus* [2]. The spillover of HeV from flying foxes appears to exclusively involve horses, with a spike in 2011 of 18 events, resulting in 24 horses succumbing to disease or requiring euthanasia. As of 2024, there have been 67 confirmed HeV outbreak incidences in Australia, including 109 horse deaths, and infection recorded in seven people, four of whom subsequently died [3].

Since HeV was recognized, a variety of serological tests have been developed. The detection of specific antibodies against HeV using the virus neutralization test (VNT) is considered the WOAH standard reference test for HeV identification [4]. However, this test can only be conducted in high-containment (PC4) laboratories and requires three to four days and highly trained laboratory staff, factors that are typically not widely available in all countries. Other available serological assays, such as the plaque neutralization assay, can be conducted in PC3 containment laboratories but first require virus-infected cell monolayers to be established using highly pathogenic live HeV under PC4 conditions, followed by fixation and virus inactivation [5,6]. More recently, a recombinant chimeric Cedar virus (rCedV)-based surrogate neutralization assay platform for HeV and Nipah virus (NiV) has been developed that is suitable for use outside high containment [6]. Here, the non-pathogenic rCedV was employed using a reverse genetics platform to generate rCedV chimeras in which the fusion (F) and glycoprotein (G) genes of CedV were replaced by those of NiV-Bangladesh and HeV, generating replication-competent chimeric viruses, and demonstrated to precisely measure the specific virus-neutralizing activities of F and G reactive monoclonal antibodies (mAb) and serum neutralization titers from either HeV or NiV-B G glycoprotein-immunized animals. 

The Australian Centre for Disease Preparedness (ACDP) laboratory in Geelong, Victoria, has both PC4 and PC3 high-containment laboratories, and routinely receives specimens for HeV exclusion testing. Clotted blood/serum, swabs and tissue samples from horses or other species are typically submitted for agent and antibody detection using molecular methods, serological assays and virus isolation. HeV serology is undertaken for disease (exclusion) investigations, surveillance and epidemiology, as well as quarantine and export testing for compliance with the certification requirements for the international movement of horses. 

At ACDP, when HeV exclusion samples have been received, the serum is treated by diluting the serum in 1/5 (*v*/*v*) in phosphate-buffered saline (PBS) containing 0.5% Tween and 0.5% Triton-X100 and then inactivated by heating at 56 °C for 30 min. This treatment allows the serology testing to be performed in PC3 laboratories rather than at PC4. Originally, HeV serology involved the use of crude HeV antigen treated with 0.1% (*w*/*v*) sodium dodecyl sulphate (SDS) in an indirect ELISA (HeV SDS iELISA; Daniels et al., 2001). This test resulted in many non-specific reactions that required confirmatory testing using the VNT conducted at biosafety PC4 containment. Subsequently, a recombinant HeV sG glycoprotein was used in an indirect sG ELISA [7,8]. The HeV sG iELISA is currently part of the Laboratories for Emergency Animal Disease Diagnosis and Response (LEADDR) network and is used by State Laboratories around Australia for HeV disease exclusions [8]. Luminex-based indirect antibody assays using HeV and NiV sG glycoproteins coupled to magnetic beads have also been developed for antibody detection and the differentiation of henipavirus infections [9]. This Luminex-based blocking assay is the first surrogate VNT developed for HeV, which importantly does not require any live cell culture or authentic virus and can be performed in a standard laboratory without any biocontainment need.

To improve the sensitivity and specificity of HeV antibody detection, a multispecies competition (blocking) ELISA, which uses a G glycoprotein-specific monoclonal antibody (mAb 1.2) [10], was developed for testing sera of horses, dogs, cats and bats [11]. This HeV sG bELISA is currently used on diagnostic serum samples received at ACDP for the detection of antibodies against HeV for the purposes of disease investigations, surveillance studies and export certification for the international movement of animals. In late 2012, a commercial equine vaccine (Equivac^®^ HeV; Zoetis, Rhodes, NSW, Australia) was launched under limited field use for the protection of horses against HeV infection. Equivac^®^ HeV was officially registered with the Australian Pesticides and Veterinary Medicines Authority (APVMA) in 2015 and, initially, horses were vaccinated with a two-dose schedule at an interval of 3–6 weeks, followed by booster vaccinations at 6-monthly intervals [12,13]. All vaccinated horses must be microchipped at the time of vaccine administration, unless already microchipped, and registered on the online registry at https://www.zoetis.com.au/vets-australia (accessed on 16 December 2024). In May 2016, the booster vaccination recommendation was adjusted to a 12-month cycle [14]. The 1 mL vaccine contains 100 µg of recombinant HeV sG glycoprotein antigen adjuvanted with 250 µg/dose of an immuno-stimulating complex and is administered by intramuscular injection. However, issues arise when equine serum samples are received at the ACDP with no accompanying record of vaccination or microchip on the submission paperwork, which causes difficulties in the interpretation of serology results. These issues are compounded by the fact that not all horses are HeV-vaccinated, with approximately only around 10–13% of horses receiving HeV vaccination during 2016–2019 in the Australian States or Territories in which HeV is endemic [14].

Since the basis of the Equivac^®^ HeV vaccine is the same recombinant-expressed HeV sG glycoprotein as used in all current serological HeV tests, a positive antibody test result cannot confirm whether this is due to a current or resolved HeV infection or due to previous vaccination, and this complicates the serology findings of such horses. Recently, the use of a different HeV protein, such as a recombinant N protein developed in yeast *Saccharomyces cerevisiae* cells [15], facilitated the development and validation of a specific HeV IgM assay for the detection of early antibody responses to HeV [16]. These findings led to the idea of using both recombinant sG and N proteins in parallel, in combination with two specific mAbs, facilitating the development and validation of the HeV DIVA ELISA.

This HeV DIVA ELISA is a major development in HeV serology and can be used with serum samples taken from horses that have been infected or vaccinated with HeV vaccine. Also, serum from horses that are non-infected or non-vaccinated with HeV can also be assessed. The intended purpose of this assay is to differentiate infected from vaccinated animals (DIVA) for HeV. The recombinant HeV sG glycoprotein detects antibodies present due to infection or vaccination when positive, and the recombinant HeV N protein detects antibodies present due to an infection when positive or due to a vaccination when negative. In this study, we undertook the validation of the HeV DIVA ELISA for analytical and diagnostic sensitivity and specificity using equine samples from four defined cohorts in Australia and demonstrated that we can serologically differentiate between HeV-infected and -vaccinated animals, thereby addressing an important gap and unmet need in HeV serological diagnostics.

## 2. Materials and Methods

### 2.1. Serum Samples

A total of 1138 serum samples were derived from clotted blood obtained from horses between 2009 and 2011. These horses represented a true negative population (HeV sG negative) because their sera were collected before the release of the HeV Equivac^®^ vaccine in 2012. These samples were designated as the negative cohort.

A total of 502 serum samples were obtained from horses previously vaccinated with Equivac^®^ HeV vaccine and submitted under the HeV DIVA project at ACDP. These horses represented a true positive population for HeV vaccination (HeV sG positive) as they all had previous vaccination and positive titers by HeV VNT. These samples were designated as the vaccinated cohort.

A total of 19 serum samples were collected from naturally HeV-infected horses from the various HeV outbreaks in Australia. These horses represented a true positive population for HeV infection (HeV sG positive) as they had positive titers by HeV VNT. These samples were designated as the positive cohort.

The control sera used for the HeV DIVA ELISA was added to columns 1 and 2 and columns 7 and 8 of each ELISA plate in duplicate. The high positive control was a 1/30 dilution of 08-02813-0001, the low positive was a 1/125 dilution of 08-02668-0001 and the negative control was Gibco horse serum (Thermo-Fisher Scientific, Scoresby, VIC, Australia).

For the analysis of analytical specificity, a panel of 25 sera (Table 1) from animals that had been previously infected with a range of paramyxoviruses, flaviviruses and alphaviruses were tested using the HeV DIVA ELISA.

For the analysis of analytical sensitivity, a HeV-infected serum (Horse #7 from the original HeV outbreak) was used. This serum sample had previously tested positive by HeV VNT, HeV sG iELISA, HeV bELISA and HeV IgM ELISA.

### 2.2. HeV DIVA ELISA

This assay utilizes the HeV soluble G tetramer antigen (HeV-sG), developed in mammalian cell lines and supplied by CSIRO Manufacturing, Clayton, Australia [7], in combination with the HeV N antigen developed in yeast cells (yHeV-N) and supplied by Rasa Petraityte-Burneikiene, Vilnius, Lithuania [15]. 

Briefly, the first half (six columns) of a 96-well flat-bottom NUNC Maxisorp ELISA immuno-plate (Thermo-Fisher Scientific, Victoria, Australia) was coated with 50 µL/well of HeV sG glycoprotein at a concentration of 0.25 µg/mL diluted in PBS. The remaining half (six columns) of the 96-well plate was coated with 50 µL/well of yeast-derived HeV N protein at a concentration of 0.33 µg/mL diluted in carbonate-bicarbonate buffer (Merck Life Science Pty Ltd., Bayswater, VIC, Australia). The ELISA plates were incubated for 1 h at 37 °C on a plate shaker with shaking. The plates were then blocked with blocking buffer (casein-blocking buffer diluted 1/10 in deionised water) and incubated for 30 min at 37 °C while shaking. The plates were washed four times with PBS containing 0.05% Tween 20 (PBST) using a plate washer (BioTek 405-TS, Millennium Science, Mulgrave, VIC, Australia). Next, 50 uL of blocking buffer was added to all wells on the plate. Then, 10 µL of control sera (high positive, low positive and negative sera) was added in duplicate to the first two columns of the G and N antigens. Also, 10 µL of test serum samples (non-triton/tween-treated) were added in duplicate, starting from columns 3 and 4 and columns 9 and 10 for both the G and N proteins, with a maximum of 16 test serum samples per plate. If a diagnostic sample is received for a HeV exclusion at ACDP, it is firstly triton/tween-treated (1/5 (*v*/*v*) dilution in PBS and 0.5% triton/tween), and then 40 µL/well of blocking buffer is removed from the well and 50 µL of the triton/tween-treated sample is added in duplicate to each well, making a total volume of 60 µL/well. The plates were incubated for 1 h at 37 °C on a plate shaker with shaking. After incubation, the monoclonal antibody (mAb) is added to the diluted serum or blocking buffer with no washing step. For the first half of the plate, 50 µL/well of anti-HeV G mAb 1.2 [10] diluted 1/500 (*v*/*v*) in blocking buffer was added to all wells except wells H1 and H2 (blank wells; 50 µL/well of blocking buffer only was added to these). For the second half of the plate, 50 µL/well of anti-NiV N mAb 1G3, produced and supplied by Indre Kucinskaite-Kodze, Vilnius, Lithuania, diluted 1/200 in blocking buffer, was added to all wells except wells H7 and H8 (blank wells; 50 µL/well of blocking buffer only was added to these). The plates were incubated for 30 min at 37 °C with shaking. Following incubation, the plates were washed as described above, and then 50 µL/well of HRP-conjugated goat anti-mouse IgG (Jackson Immuno Research Laboratories, Cannon Hill, QLD, Australia), diluted 1/2000 (*v*/*v*) in blocking buffer, was added to all wells on the plate. The plates were further incubated for 30 min at 37 °C while shaking. Then, 50 µL/well of TMB liquid substrate (Merck Life Science Pty Ltd., Victoria, Australia) was added to all wells on the plate. The plates were incubated at room temperature for approximately 10 min (monitored by negative control wells reaching 0.35 OD using a 650 nm wavelength), and then 50 µL/well of 1M H_2_SO_4_ (Ajax Finechem, Taren Point, NSW, Australia) was added to all wells on the plate. The plates were read at 450nm with a Multiskan FC Microplate Photometer (Thermo-Fisher Scientific, Victoria, Australia). The percentage inhibition of the samples was calculated using the OD values obtained for their respective HeV G and N controls, using the following formula: % inhibition = 100 × (1 − (OD test serum/OD negative control)). Results were reported as follows: (i) G -N-: “negative”; (ii) G+N-: “consistent with vaccination”; or (iii) G+N+: “consistent with infection”. If results are G -N+:, additional testing is required and suspected exposure or disease investigations should be followed up with a re-bleed collected 7 to 14 days following the initial sample. Also, the sample should be tested in the HeV VNT and HeV IgM ELISA to assist with the interpretation of this result.

## 3. Results

### 3.1. Analytical Sensitivity (ASe)

**Figure 1:**
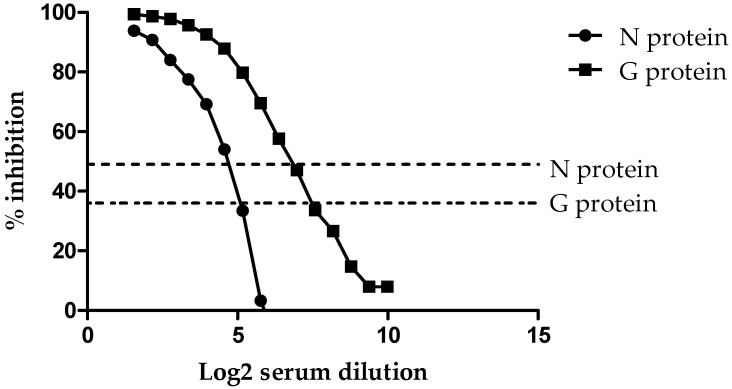
Titration of the positive control for the HeV DIVA ELISA with % inhibition (Y axis) in relation to the serum dilution (X axis). Both the N protein and G glycoprotein threshold lines are represented by dotted lines.

To demonstrate the analytical sensitivity of the HeV DIVA ELISA, a horse naturally infected with HeV (Horse #7 from the original HeV outbreak) was titrated two-fold across the plate for both the G and N proteins. As shown in Figure 1, using a cut-off value of 36% inhibition for the sG glycoprotein, the sample tested positive out to the 1/6144 dilution. 

However, when using the cut-off value of 49% inhibition for the N protein, the HeV positive sample was positive out to the 1/192 dilution. This indicates that the sG glycoprotein is 32 times more sensitive than the N protein when read at their respective cut-off values. In addition, this same horse sample was titrated using the HeV VNT and found to be positive with a titer of 1600.

### 3.2. Analytical Specificity (ASp)

To determine the analytical specificity of the HeV DIVA ELISA, a panel of 25 sera with antibodies against a range of paramyxoviruses, flaviviruses and alphaviruses was examined. As shown in Table 1, testing using the sG glycoprotein resulted in all sera being below the cut-off value of 36% inhibition and thus negative for the sG glycoprotein.

**Table 1:** Detection of HeV antibodies in a panel of 25 sera from a range of paramyxoviruses, flaviviruses and alphaviruses using the HeV DIVA ELISA and a standard serum dilution.

Antisera	G Protein (% Inhibition)	N Protein (% Inhibition)
Anti-PPR (Caprine)	32	−7
Anti-Rinderpest (Rabbit)	18	−29
Anti-Measles (Human)	−2	−37
Anti-Canine Distemper Virus (Horse)	−3	−25
Anti-NDV V4 (Rabbit)	26	−14
Anti-J Virus (Rabbit)	15	4
Anti-Parainfluenza Type 1 (Horse)	10	−1
Anti-Parainfluenza Type 2 (Horse)	−10	−46
Anti-Parainfluenza Type 3 (Horse)	4	−24
Anti-Parainfluenza Type 1 (Guinea pig)	24	−28
Anti-Parainfluenza Type 4A (Guinea pig)	5	−14
Anti-Parainfluenza Type 4B (Guinea pig)	23	−30
Anti-Mumps Enders Strain (Horse)	3	−11
Anti-Nariva (Rabbit)	11	−32
Anti-Tioman Virus (Pig)	35	−19
Anti-Menangle Virus (Rabbit)	10	−34
Anti-Menangle Virus (Pig)	29	−14
Anti-Blue Eye Rubulavirus (Pig)	−8	−10
Anti-Mossman Virus (Rabbit)	16	−18
Anti-Japanese Encephalitis (Pig)	3	−22
Anti-Murray Valley Encephalitis Virus (Pig)	8	26
Anti-West Nile Virus (Horse)	10	2
Anti-African Horse Sickness Virus (Horse)	13	−9
Anti-Canine Distemper Virus (Horse)	8	−27
Anti-Hendra Virus (Horse)	99	94

It should be noted that the anti-Tioman virus (Pig) sera had a result of 35% inhibition using the sG glycoprotein, which is close to the cut-off <36% inhibition. Tioman virus is in the family *Paramyxoviridae* and was first discovered in 2001 when analyzing urine from pteropod bats (flying foxes; *Pteropus hypomelanus*) found on Tioman island off the eastern coast of Malaysia [17]. When assessing the N protein, all sera yielded a signal below the cut-off value of 49% inhibition and thus a negative result. These results were then compared to a horse naturally infected with HeV, which tested positive with 98.56% inhibition for the sG glycoprotein and positive with 94.27% inhibition for the N protein, giving a result of G+N+, and thus consistent with authentic HeV infection.

### 3.3. Diagnostic Specificity (DSp)

**Figure 2:**
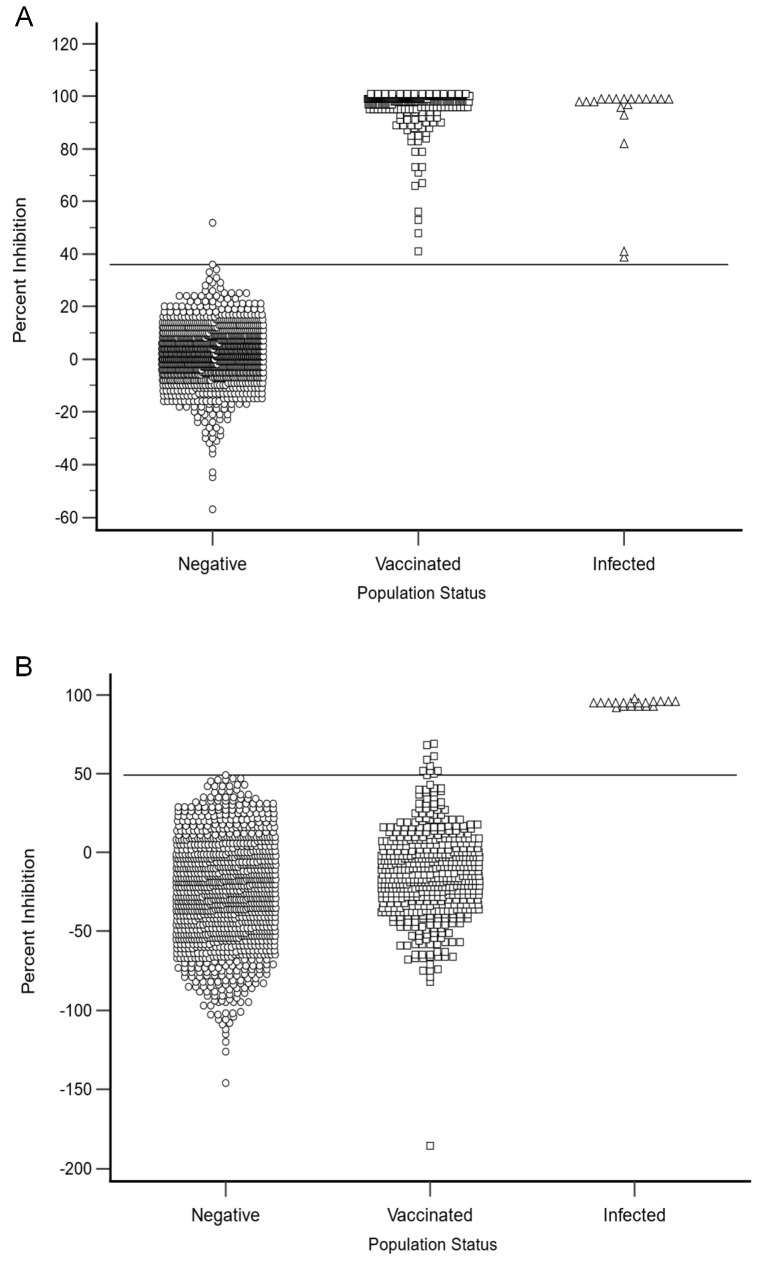
The HeV DIVA ELISA percent inhibition results for the negative, vaccinated and positive (infected) equine cohorts are shown for both the sG (**A**) and N (**B**) proteins. The sG and N protein ELISA thresholds are represented by solid lines. A percentage inhibition threshold value of >36 for the sG glycoprotein ELISA resulted in a DSe of 100.0% (95% CI: 99.3–100.0%) and a DSp of 99.9% (95% CI: 99.5–100.0%), while for the N protein, a percentage inhibition cut-off value of >49 resulted in a DSe of 100.0% (95% CI: 82.4–100.0%) and a DSp of 100.0% (95% CI: 99.7–100.0%).

To determine the diagnostic specificity of the HeV DIVA ELISA, three different cohorts of horses were examined, namely, 1138 negative cohort sera, 502 vaccinated cohort sera and 19 positive cohort sera. Using the combined inhibition values for the positive, vaccinated and negative cohorts, receiver operating characteristic (ROC) graphs plotting DSe against 100-Dsp were used to optimize the selection of thresholds for the G and N protein in the HeV DIVA ELISA. As shown in Figure 2 and Table 2, DSe and DSp were optimized when the positive threshold (cut-off value) for the G protein was set at a value of >36% inhibition; this gave a DSe of 100.0% (95% CI: 99.3–100.0%) and a DSp of 99.9% (95% CI: 99.5–100.0%). Using the same method of producing an ROC graph, the positive threshold (cut-off value) for the N protein was set at >49% inhibition, which resulted in a DSe of 100.0% (95% CI: 82.4–100.0%) and a DSp of 100.0% (95% CI: 99.7–100.0%).

When assessing the negative cohort sera using the sG glycoprotein, there were only two sera higher than the cut-off value of <36% inhibition, namely, 10-01954-0006 with 36% inhibition and 11-00356-0020 with 52% inhibition. With the N protein, there was one sample higher than the <49% cut-off value, namely, 11-00367-0001 with 49% inhibition. Two of these results were extremely close to the cut-off values using the sG glycoprotein and the N protein and therefore were not significant positive results. Other than these three sera, all other negative cohort sera were below the cut-off values, and this demonstrates the very low numbers of non-specific reactors when using this test.

### 3.4. Diagnostic Sensitivity (DSe)

To determine the diagnostic sensitivity of the HeV DIVA ELISA, 19 samples representing the positive cohort sera were tested, and all sera were positive using the sG glycoprotein, confirming 100% DSe (95% CI: 99.3–100.0%), as shown in Table 3. 

All sera were also positive using the N protein with DSe 100.0% (95% CI: 82.4–100.0%), which indicates G+N+, “consistent with infection”. In addition, these sera were all positive by HeV VNT, as shown in Table 3.

### 3.5. Repeatability

To assess the repeatability of the HeV DIVA ELISA, two operators tested three different horse sera over 12 different tests in the same laboratory. The three sera were (1) a horse naturally infected with HeV, (2) a horse vaccinated with Equivac^®^ HeV vaccine and (3) a horse uninfected with HeV and non-vaccinated with HeV vaccine.

The results in Table 4 show that the three horses had been correctly identified over the 12 separate tests using the HeV DIVA ELISA. 

The low standard deviation results of 0.5 (for the sG glycoprotein) and 0.8 (for the N protein) for the infected horse showed good repeatability when testing horses naturally infected with HeV. The vaccinated horse had a low standard deviation of 1.1 (for the sG glycoprotein) and a higher standard deviation of 14.9 (for the N protein), indicating greater variation when testing using the N protein (ranging from −63.2 to −1.7% inhibition). However, it should be noted that the N protein was always in the negative range and not close to the cut-off value for this assay. Much higher standard deviations of 14.4 (for the sG glycoprotein) and 14.8 (for the N protein) were obtained for the uninfected and non-vaccinated horse, but once again the percent inhibition values were all well under the cut-off values for these assays.

### 3.6. New Algorithm for HeV Serology Testing at ACDP

**Figure 3:**
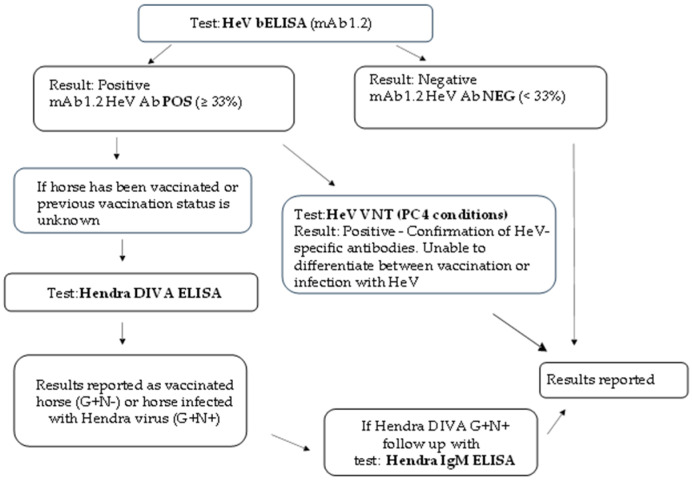
New algorithm diagram for HeV serology at ACDP.

A new algorithm pathway for HeV serological testing is now followed for all diagnostic serum samples received at ACDP, as shown in Figure 3.

This also highlights the importance of the HeV DIVA ELISA strategy for antibody detection and its role for the differentiation of Equivac^®^ HeV-vaccinated horses from HeV-infected horses. Regardless, multiple HeV serological tests are conducted to determine the infection status of the serum sample. In most circumstances, swab and/or tissue samples from the same animal are submitted to further investigate the animal’s disease status using molecular (PCR) and/or virology (isolation) methods.

## 4. Discussion

Presently, the HeV-specific VNT is considered the ‘gold standard’ assay for detecting HeV-specific antibodies that can neutralize the virus. However, this test requires the use of live HeV, a high-security biosafety level 4 (PC4) laboratory, and three–four days to obtain the results. Thus, the use of ELISAs provides a simple rapid procedure requiring only small volumes of sera, is not as labor-intensive nor time consuming for large-scale or high-throughput serological testing, and can be conducted in a lower-security PC3 laboratory setting. Since the discovery of HeV, the serological tests have progressed from initially using crude viral antigen preparations from infected cells to the use of purified recombinant viral antigens. Furthermore, the ELISA format has evolved from indirect ELISAs to the use of more specific competition ELISAs which include a specific mAb, providing increased sensitivity and specificity as well as their application to multiple animal species. Here, we detail the development and use of a HeV DIVA ELISA using two recombinant proteins (HeV G and N) and two specific mAbs; this assay is the first HeV DIVA competitive ELISA that can differentiate between Equivac^®^ HeV-vaccinated and HeV-infected horses in Australia. The DIVA ELISA approach has been applied to other viruses with the use of vaccination in animals following a disease outbreak and has been successful with pseudorabies and avian influenza eradication [18]. The DIVA has been proposed for Foot and Mouth Disease virus (FMDV) eradication using the PrioCHECK FMDV NSP ELISA, which detects antibodies against the conserved 3ABC non-structural protein. This assay can identify FMDV infection across all serotypes and distinguishes infected from vaccinated animals, detecting FMDV in cattle, sheep, goats or pigs regardless of the strain [19,20]. 

The present study also supports that Equivac^®^ HeV vaccination can provide protection against an HeV infection in horses by the assessment of HeV-specific antibodies in the HeV DIVA ELISA with the 502 vaccinated serum samples. In addition, the entire cohort of vaccinated sera had positive antibody titers detected in the HeV VNT. Indeed, several studies have shown that the Equivac^®^ HeV vaccination protocols within Australia are capable of eliciting HeV-specific neutralizing antibody responses confirmed by the HeV VNT in horses, and they are critical in managing the risk of a HeV spillover or transmission of infection among the Australian horse population [14,21]. 

A recent study involved the development and evaluation of a competitive ELISA for the serodiagnosis of NiV and HeV infection using a recombinant NiV G glycoprotein and a NiV G-specific monoclonal antibody [22]. This NiV cELISA is a major improvement in using a recombinant NiV G glycoprotein instead of the current indirect NiV ELISA at ACDP which uses whole live crude antigen preparations under PC4 conditions [23]. This further provides evidence for the improvement in sensitivity and specificity when using recombinant proteins instead of crude antigens in an ELISA format, as shown previously with an HeV sG competition compared to an indirect HeV recombinant soluble G ELISA or an indirect whole crude antigen HeV ELISA at ACDP [8,11,23]. Also, the NiV cELISA was shown to detect antibodies against both NiV and HeV in different animal species [22]. However, this NiV cELISA also identifies specific antibodies to the G protein, which is the protective antigen used in the HeV vaccine and therefore can only be used for post-vaccination monitoring to determine seroconversion. Thus, this NiV cELISA has no ability to be used in a DIVA format, unlike the HeV DIVA ELISA described in our study. 

Previously, this HeV DIVA ELISA was used as a reference test to help in validating an indirect ELISA (FLI HeV DIVA ELISA) produced in Germany by using G proteins expressed in *Leishmania tarentolae* and N proteins expressed in recombinant baculovirus-infected insect cells [24]. Some problems arose with the indirect FLI HeV DIVA ELISA, as it used a horse-specific conjugate, so it could only be used for testing horses, and being an indirect assay, it had increased non-specific reactors, which resulted in lower diagnostic specificity for the G (99.4%) and N (99.3%) proteins compared to the HeV DIVA ELISA at ACDP.

The present study demonstrates that the HeV DIVA ELISA strategy detailed here, which uses competition ELISAs based on recombinant protein sG or N and two virus-specific mAbs, results in improved diagnostic sensitivity and diagnostic specificity. This HeV DIVA ELISA had improved statistical results, with 99–100% diagnostic sensitivity for both the sG and N proteins when using their respective cut-off values. Previously, an HeV sG competitive ELISA was shown to have improved sensitivity and specificity compared to an indirect HeV sG ELISA [11]. Another disadvantage of an indirect ELISA is the need to change the secondary antibody depending on the animal species or human being tested. Further, both HeV and NiV are zoonotic viruses and can infect several animal species such as horses, dogs, cats and pigs, in addition to humans, so a competitive ELISA is more suitable for testing multiple species and humans as compared to an indirect ELISA.

Equine serum samples, which have returned a positive result in the HeV sG indirect ELISA at State Animal Health Laboratories around Australia, arrive at the ACDP monthly for further analysis. On some occasions, the status of the horse’s HeV Equivac^®^ vaccination history is unknown. Thus, the HeV DIVA ELISA described in this study is the only serological test which can determine if this positive result is due to a non-specific reaction or a true infection with HeV or because of Equivac^®^ HeV vaccination. This new testing algorithm for HeV serological testing at ACDP will facilitate the correct identification and immune status of the horses being assessed. The HeV DIVA ELISA detailed here has now been validated and is a new and valuable tool, filling a previously unmet need to facilitate the correct determination of the HeV-specific immune status of horses.

## 5. Patents

The author Christopher C. Broder is an United States federal employee and co-inventor on US and foreign patents pertaining to soluble forms of the Hendra virus and Nipah virus G glycoproteins whose assignee is the United States as represented by the Henry M. Jackson Foundation for the Advancement of Military Medicine, Inc. Soluble forms of the Hendra virus and Nipah virus G glycoproteins are licensed to Zoetis Inc. This work was performed under an MTA agreement (effective from 1 May 2008) between CSIRO and The Henry M. Jackson Foundation for the Advancement of Military Medicine, Inc. for the mutual exchange of biological research materials and confidential information.

## Figures and Tables

**Table 2:** The summary of diagnostic specificity and sensitivity results using the HeV DIVA ELISA.

Variable	N Assay	G Assay
Infected	19	19
Vaccinated	502	502
Negative	1138	1138
Cut-off	<49	<36
DSe	100%	100%
DSp	100%	99.91%

**Table 3:** Assessment of the 19 naturally HeV infected field sera using the HeV DIVA ELISA and the HeV VNT. The assay positive detection thresholds are HeV DIVA ELISA % inhibition >36 for the G protein and % inhibition >49 for the N protein; HeV VNT positive ≥ titer of 2.

Sample IDs	G Protein	Result	N Protein	Result	HeV VNT	Result
08-02438-0027	97.29	Positive	95.81	Positive	2048	Positive
08-02480-0001	97.87	Positive	95.46	Positive	4096	Positive
08-02667-0002	98.61	Positive	95.16	Positive	2048	Positive
08-02669-0001	98.73	Positive	92.98	Positive	2048	Positive
08-02668-0001	98.56	Positive	94.68	Positive	2048	Positive
08-02844-0001	41	Positive	94.57	Positive	16	Positive
08-02813-0001	98.49	Positive	92.33	Positive	512	Positive
06-03803-0004	39	Positive	93.12	Positive	20	Positive
09-02723-0001	92.89	Positive	93.1	Positive	1024	Positive
09-02723-0002	96.08	Positive	92.72	Positive	16	Positive
09-02844-0007	98.9	Positive	94.89	Positive	>16	Positive
09-02844-0008	98.74	Positive	94.67	Positive	640	Positive
163910/1	99.36	Positive	95.74	Positive	640	Positive
163910/2	98.99	Positive	95.79	Positive	640	Positive
163910/3	98.2	Positive	97.82	Positive	1280	Positive
163910/4	81.78	Positive	93.05	Positive	20	Positive
163910/5	99.22	Positive	94.71	Positive	640	Positive
163910/6	99.12	Positive	94.98	Positive	640	Positive
163910/7	99.48	Positive	95.8	Positive	640	Positive

**Table 4:** Summary statistics for the repeatability of the HeV DIVA ELISA using three sera from the positive (1), vaccinated (2) and the negative (3) cohorts of horse sera over twelve different tests.

	Positive (1)		Vaccinated (2)		Negative (3)	
	G	N	G	N	G	N
Count	12	12	12	12	12	12
Average PI	98.9	98.6	99.9	−23.6	12.7	−18.7
Standard deviation	0.5	0.8	1.1	14.9	14.4	14.8
Maximum PI	99.7	99.6	101.3	−1.7	32.1	−1.8
Minimum PI	97.9	97.3	97	−63.2	−12.9	−54.6
Median PI	98.9	98.7	100.1	−19.5	10.6	−15
Results	G+	N+	G+	N-	G-	N-

## Data Availability

All data are in results section.

## Abbreviations

The following abbreviations are used in this manuscript:

DSe	Diagnostic sensitivity
DSp	Diagnostic specificity
ASe	Analytical sensitivity
ASp	Analytical specificity
DIVA	Differentiating infected from vaccinated animals
ELISA	Enzyme-linked immunosorbent assay
VNT	Virus neutralization test
HeV	Hendra virus
